# From Folk Medicine to Pharmacology: A Systematic Review of the Anti-Inflammatory Evidence for *Hymenaea* spp. (Fabaceae)

**DOI:** 10.3390/plants14223545

**Published:** 2025-11-20

**Authors:** Joy Braga Cavalcante, Adriely de Brito Silva, Roberta de Freitas Lopes, Lucimar Pinheiro Rosseto, Fabiana Aparecida Marques, Osmar Vieira da Silva, José Luís Rodrigues Martins, João Maurício Fernandes Souza, Lucas Barbosa Ribeiro de Carvalho, Natascha de Vasconcellos Otoya, Sandro Dutra e Silva, Iransé Oliveira-Silva, Luis Eduardo Maggi, Rogério de Freitas Lacerda, Elson Alves Costa, Leonardo Luiz Borges, James Oluwagbamigbe Fajemiroye

**Affiliations:** 1 Department of Biotechnology, Xapuri Campus, Federal Institute of Education, Science and Technology of Acre (IFAC), Rio Branco 69930-000, AC, Brazil; 2 Center of Health and Sports Sciences, Federal University of Acre (UFAC), Rio Branco 69906-310, AC, Braziljamesfajemiroye@ufg.br (J.O.F.); 3 Graduate Program, Evangelical University of Goiás (UniEVANGÉLICA), Anápolis 75083-515, GO, Brazil; 4 Chemistry Department, Federal Institute of Education, Science and Technology of Goiânia (IF Goiano), Ceres 76300-000, GO, Brazil; 5 School of Medical and Life Sciences, Pontifical Catholic University of Goiás, Goiânia 74605-010, GO, Brazil; lubarica@gmail.com; 6 Central Campus, State University of Goiás, Anápolis 75132-903, GO, Brazil; 7 Center of Biological and Natural Sciences, Federal University of Acre (UFAC), Rio Branco 69906-310, AC, Brazil; luis.maggi@ufac.br (L.E.M.);; 8 Department of Pharmacology, Federal University of Goiás (UFG), Goiânia 75083-515, GO, Brazil; xico@ufg.br

**Keywords:** *Hymenaea*, inflammatory diseases, herbal medicine, phytochemicals, ethnopharmacology, plant extracts

## Abstract

*Hymenaea* spp. (Fabaceae) are widely used in folk medicine to treat fatigue, inflammation, respiratory, and gastrointestinal disorders. However, comprehensive evidence-based evaluations of their pharmacological potential remain limited. This systematic review brought together the existing pharmacological and phytochemical evidence on the significant therapeutic potential of *Hymenaea* spp. A total of 17 studies were included; phytochemical analyses identified flavonoids, triterpenes, procyanidins, xyloglucans, and caryophyllene oxide among the major bioactive constituents. The reported biological activities were primarily anti-inflammatory, antioxidant, immunomodulatory, antimicrobial, and antiproliferative. Mechanistic findings consistently substantiated anti-inflammatory evidence through COX/LOX inhibition, cytokines, and redox-related modulations. Despite these promising results, the molecular mechanisms and translational evidence remain poorly defined. In conclusion, *Hymenaea* spp. exhibit significant pharmacological potential. Future studies integrating metabolomics and preclinical and clinical validation are essential to translating traditional knowledge of this species into evidence-based therapeutics.

## 1. Introduction

Silent witnesses to millions of years of ecological evolution, *Hymenaea* spp. (Fabaceae) are predominantly neotropical trees with a deep environmental history embedded in their foliage. Fossilized amber unearthed in the Caribbean suggests that members of this genus reached the American tropics approximately 15 million years ago, with strong evidence of an African origin preceding their diversification in the Americas [1]. Today, the various species of *Hymenaea* thrive across a vast geographic range, from southern Mexico to Brazil and the Caribbean, with cultivation extending into parts of Asia. In Brazil, for example, botanical and historical records confirm the presence of this species across various regions and biomes, including the tropical forests of the Brazilian coast and interior [2,3]. This long evolutionary trajectory has woven *H.* spp. into the cultural fabric of numerous societies, particularly in tropical regions of Latin America, where they are integral to traditional medicine practiced by Indigenous, riverine, and rural populations. Commonly used to treat respiratory infections, joint pain, inflammation, and gastrointestinal disorders, mainly *H. courbaril* L., *H. stigonocarpa*, *H. rubriflora*, and *H. martiana*, their leaves, seeds, bark, and resin are often prepared as balms or extracts, reflecting a broad spectrum of empirically established applications [4,5,6,7]. Historically, the use of plants in the treatment and cure of diseases is as old as the human species itself, ref. [8] and modern science has increasingly focused on compounds present in plants with therapeutic potential, especially secondary metabolites, which are associated with plant survival and propagation and may present highly relevant pharmacological properties [9]. *H.* spp., belonging to the subfamily Caesalpinioideae of the Fabaceae family, are an emblematic example of plants with both a broad ethnobotanical tradition and notable pharmacological importance.

Advances in ethnopharmacological research and modern phytochemical techniques have revealed a variety of bioactive compounds in *H.* spp., including flavonoids such as quercetin, rutin, and astilbin, along with tannins, terpenes, sesquiterpenes, and proanthocyanidins [10,11]. These metabolites have been linked to anti-inflammatory, ref. [6] antimicrobial, ref. [7] antifungal, ref. [12] immunomodulatory, ref. [13] and antioxidant activities, ref. [14] most of which have been demonstrated primarily in vitro. Relatively few studies have advanced to in vivo models, and mechanistic insights into the molecular targets of isolated constituents remain limited. In parallel, popular use of “Selva”, a naturally occurring brownish, wine-like extract, as a tonic for endurance, fatigue relief, and sexual vigor is relatively common, yet scientific evidence supporting these effects is still scarce and largely anecdotal.

**Figure 1:**
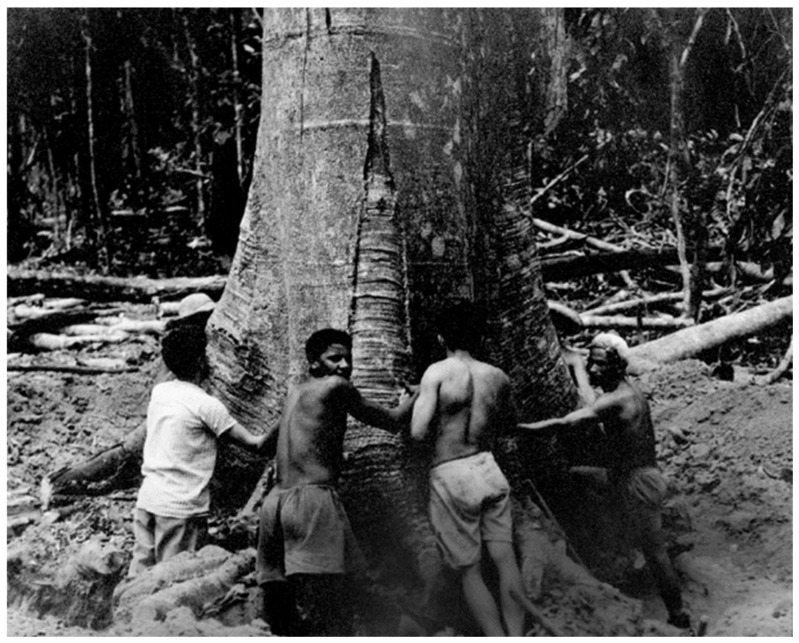
A group of “mateiros” (woodcutters) embracing a tree (*H.* spp.). 2 February 1959. Source: Public Archive of the Federal District—ArPDF.

Among *H.* spp., *H. courbaril* stands out as a neotropical species with deep ethnopharmacological roots across Latin America [15,16,17]. The resin, known as “animé,” has historically been used both medicinally and ritually, burned in sacred contexts, and valued for its antiseptic and aromatic properties [18,19]. Beyond its ethnomedical significance, *H.* spp. has also carried symbolic weight in Brazil’s political and environmental history [20] (Figure 1).

Pharmacological research on *H.* spp. remains fragmented, with limited integration among phytochemical, pharmacodynamic, and ethnobotanical approaches. As a result, much of the therapeutic potential attributed to *H.* spp., such as anti-inflammatory, antioxidant, antimicrobial, and analgesic effects, still requires rigorous scientific validation, particularly regarding less-studied properties such as anticancer, cardioprotective, and neuroprotective effects [10,13,21,22]. Moreover, variability in extraction methods, plant parts utilized, and the geographical origin of samples has hindered cross-study comparisons and compromised reproducibility. For example, research on *H. courbaril* has shown distinct chemical profiles among leaves, bark, and resin, each containing different groups of compounds that directly influence the observed pharmacological effects [14,19]. Although chromatography is often employed to identify the majority of flavonoids, complete methodological standardization remains an essential but still preliminary step in advancing this field.

When seeking information on *H.* spp., there is a notable scarcity of systematic research correlating the phytochemical profiles of different species with specific, underexplored pharmacological activities—such as anticancer, cardioprotective, and neuroprotective effects. Furthermore, few investigations have examined the molecular mechanisms of action of these compounds, and studies employing in vivo protocols or standardized clinical trials remain largely absent [23,24]. These limitations underscore a critical knowledge gap that must be addressed if the pharmacological promise of *H.* spp. is to be translated into clinically relevant applications. Considering these gaps, the present systematic review was designed to critically evaluate the available evidence on the pharmacological properties of *H.* spp., focusing on identified bioactive compounds and their potential associations with emerging therapeutic applications.

## 2. Results

### 2.1. Botany and Ethnobotany

**Figure 2:**
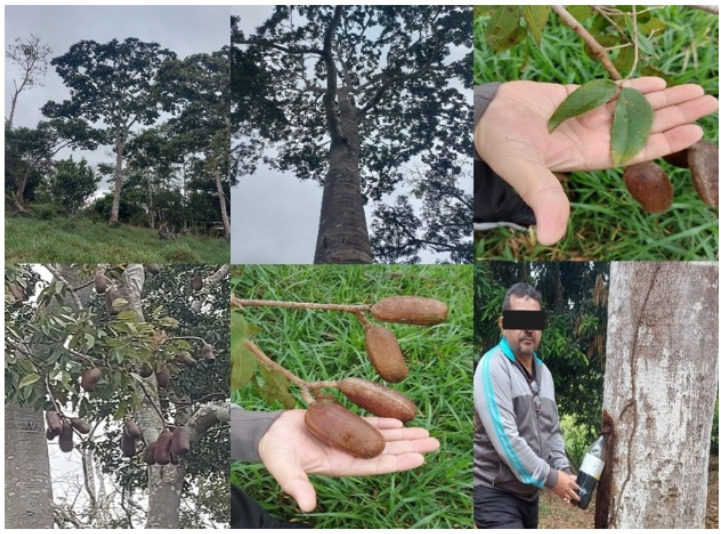
Illustrations showing *H. courbaril* trees, leaves, fruits, and sap collection. Source: Joy Braga Cavalcante.

The morphology of plants belonging to *H.* spp., specifically *H. courbaril*, is depicted in Figure 2, showing stems, flowers, and fruits. *H. courbaril* is a tree species native to the Chico Mendes Extractive Reserve (Resex Chico Mendes), a conservation unit in the southwestern region of Acre state, Brazil, covering 970,570 hectares. This reserve supports a forest with high biological diversity, and its local economy is based mainly on plant extraction [25,26]. The species is well known among extractivists and is regarded as rare, exhibiting naturally low population density [27]. Mature individuals typically reach heights of 30–40 m, with a straight trunk up to 2 m in diameter. The bark is grayish to dark brown, with deep longitudinal fissures [27]. *H. courbaril* bears compound, alternate, petiolate, bifoliate leaves with a leathery texture, typically sickle-shaped or oval. Inflorescences occur in terminal panicles, and the indehiscent woody legume fruits are green when immature, dark brown when ripe, and black when senescent, measuring 8–15 cm in length. Each fruit contains 2–6 or more seeds surrounded by a starchy, edible flour of high nutritional value, which is a vital food resource for economically disadvantaged human populations and also consumed by rodents [28,29].

Ethnobotany is an interdisciplinary field that examines the knowledge, cultural significance, management, and traditional uses of plant resources [30]. These studies extend beyond the conventional scope of botany, emphasizing the cultural value of plants within specific human communities [31]. The contribution of traditional peoples to the exploration and stewardship of natural environments is undeniable, offering insights into diverse strategies for managing and utilizing plants in daily life-resources that often represent the only available therapeutic options [32]. This body of ancestral knowledge, transmitted across generations, forms an invaluable intangible heritage essential to safeguarding the health and cultural identity of Indigenous peoples, riverine populations, quilombolas, and other traditional communities [33]. Modern ethnobotany recognizes this traditional knowledge as a key tool in the search for new phytotherapeutics and bioactive compounds, particularly when integrated with multidisciplinary scientific research in phytochemistry and pharmacology [34]. This approach is especially significant in highly biodiverse countries such as Brazil, where the interplay between biodiversity and traditional knowledge offers a unique opportunity for sustainable development and the valorization of popular culture [35].

The genus *Hymenaea* serves as a prime example of this synergy between cultural tradition and scientific validation. Across its geographic distribution, various species have been integral to folk medicine, with their uses meticulously documented through ethnobotanical studies. For instance, the resin is widely used as an anti-inflammatory and healing agent, applied topically to wounds, skin ulcers, and rheumatic joints. Internally, decoctions and infusions made from the bark and leaves are commonly employed to treat respiratory ailments such as bronchitis and asthma, as well as gastrointestinal issues, including ulcers and diarrhea. The sap is also consumed directly to combat fatigue and as a general tonic, reflecting its perceived restorative properties [36].

*H.* spp. are widely distributed across Brazilian tropical biomes, especially in the Cerrado, where they represent arboreal elements of high ecological, medicinal, and cultural importance. In a floristic survey conducted at the Biological Reserve of the Santa Rita Experimental Farm in Minas Gerais, *H. stigonocarpa* Mart. ex Hayne was recorded among the main native species used for medicinal purposes, belonging to the Fabaceae family, notable for its diversity of bioactive secondary metabolites such as tannins, flavonoids, and diterpenes [37]. The study reports the use of the bark and resin of the “jatobá-do-cerrado” in popular preparations aimed at treating anemia, inflammation, wounds, and metabolic disorders, in addition to its application as a tonic restorative. These records corroborate the broad, widespread acceptance of the species as a traditional herbal medicine, reinforcing its ethnobotanical and pharmacological relevance within the context of medicinal plants of the Cerrado.

In the traditional community of Conceição-Açu, in Mato Grosso, *H. stigonocarpa* also ranks among the species with the highest use value (VU = 2.33), being cited for the treatment of anemia, weakness, inflammation, and blood-related diseases, and applied as a sap or latex known locally as “jatobá blood” [38]. This reddish exudate, used both as a tonic and in symbolic rituals, is culturally associated with vitality and bodily purification, representing a link between physical health and spirituality. Beyond its therapeutic uses, the jatobá provides durable wood and edible fruits, integrating sustainable practices of subsistence and healing within Cerrado communities. The convergence of medicinal use, cultural symbolism, and ecological management confirms *H.* spp. as a key species in Brazilian ethnobotany, whose traditional valorization constitutes an essential foundation for contemporary phytochemical and pharmacological studies.

Beyond their ethnobotanical role, the genus *Hymenaea* has also been investigated in broader botanical and paleobotanical contexts. Comprising approximately 16 species distributed from Mexico to South and Central America, of which occur in Brazil, the genus is widely studied in both scientific and traditional medicinal contexts [39,40]. Paleobotanical studies indicate that *H.* spp. originated in African equatorial forests and reached the American continent via oceanic dispersal in the early Tertiary, resulting in the current amphi-Atlantic distribution [41,42]. At present, *H. verrucosa* remains the sole African representative of the genus. At the same time, species such as *H. courbaril*, *H. stigonocarpa*, and *H. martiana* are highly relevant in Brazil, being widely utilized in civil construction, urban landscaping, and folk medicine [29].

A closer examination of these folk applications reveals a rational basis for their use, often aligning with the pharmacological properties later identified in laboratory studies. The anti-inflammatory use of the resin, for example, is supported by the presence of terpenoids like caryophyllene oxide. Similarly, the employment of bark and leaf preparations for infections is consistent with the demonstrated antimicrobial activity of flavonoids such as astilbin and tannins. This correlation underscores that traditional knowledge is not merely anecdotal but is often based on long-term, empirical observation of a plant’s efficacy, providing a highly valuable starting point for targeted scientific investigation [43].

In addition to their medicinal value, *H.* spp. also possess significant economic importance: the resin is employed in the production of varnishes and incense, the wood is known for its durability and is used in shipbuilding, and the seeds yield edible flour incorporated into popular cuisine [44]. Regarding ethnopharmacological applications, the sap of *H. courbaril* is traditionally consumed in its natural form to treat fatigue, worm infestations, and gastrointestinal, respiratory, and urinary disorders [45]. The resin is valued as a healing agent, anti-inflammatory, and flavoring component in traditional rituals. At the same time, leaves, fruits, seeds, and even the entire plant are used to treat conditions ranging from diabetes and prostatitis to infectious and rheumatic diseases [46]. Thus, the ethnobotany of *H.* spp. transcends the mere cataloging of popular uses, revealing a deep interconnection between biodiversity and traditional knowledge. This integrative perspective not only highlights strategies for flora conservation, sustainable resource management, and preservation of cultural heritage but also underscores the potential of *H.* spp. as a model for aligning traditional knowledge with modern pharmacological discovery [29].

### 2.2. Study Selection

The *H.* spp. found in searches carried out in the electronic databases Medline/PubMed, Web of Science, Scopus, and SciELO are presented, detailing the species name, traditional use, traditional preparation for consumption, dosage, study type, experimental details, and prominent authors, as shown in Table 1.

**Figure 3:**
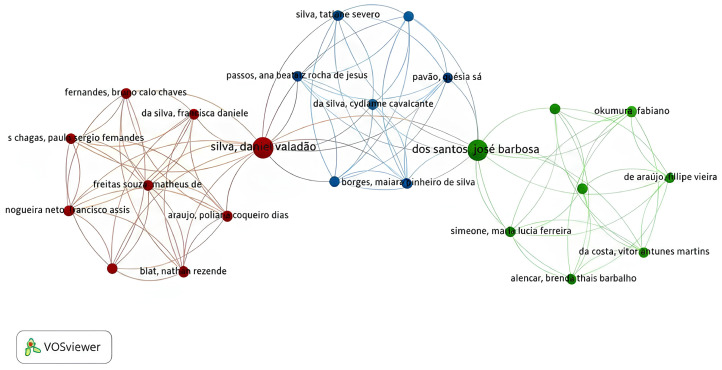
Network map of the co-occurrence matrix, showing the authors of studies on *H.* spp.

**Figure 4:**
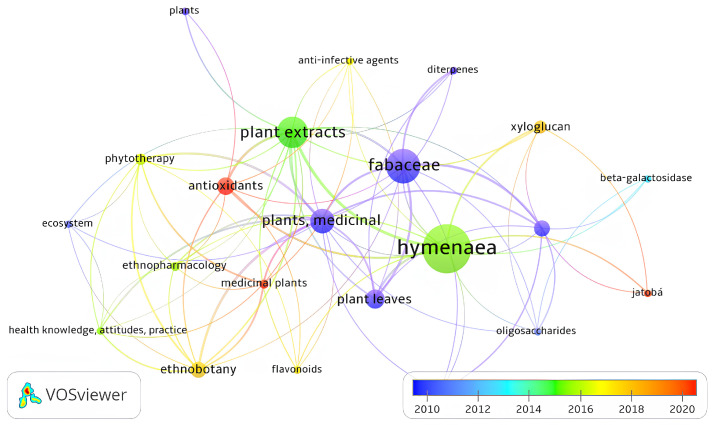
Network map of the co-occurrence matrix of keywords from studies on *H.* spp.

The co-occurrence analysis revealed that, among the most relevant contributors, Silva, Daniel Valadão occupies a central position in the network, linking the red and blue clusters, while dos Santos, José Barbosa connects the blue and green clusters. Keyword mapping demonstrated a temporal shift in research focus: around 2015, studies predominantly featured *Hymenaea*, plant extracts, phytotherapy, and ethnopharmacology, whereas, after 2020, the terms antioxidants, medicinal plants, and Jatobá emerged with greater prominence, as shown in Figure 3 and Figure 4, respectively.

**Figure 5:**
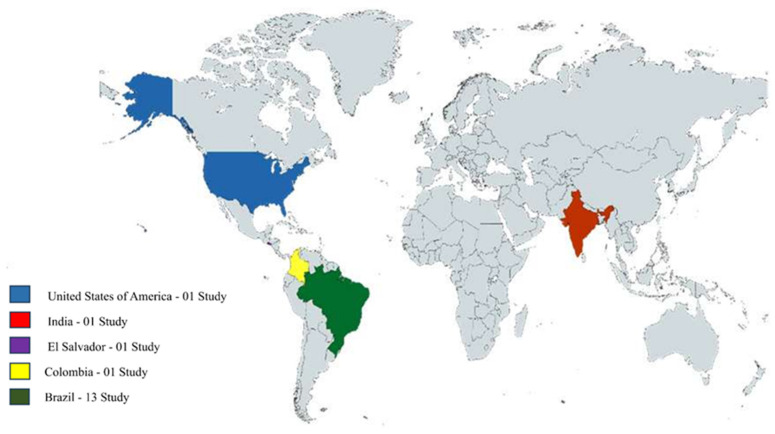
Geographical and quantitative distribution of countries with studies on the pharmacological properties of *H.* spp. included in this review.

The articles included in this review were published between 2002 and 2024, with no clear predominance of annual output; however, two were published in 2007, three in 2013, and two in 2016. Regarding the transparency of data reporting, eleven studies declared financial support from research institutions, two from government agencies, three did not report any financial support, and one indicated funding from both a government agency and private equity firms. Most of the included studies were conducted in Brazil, totaling thirteen, while Colombia, El Salvador, India, and the United States of America each contributed a single article. The predominance of Brazilian studies underscores the country’s leading role in research on *H.* spp. Notably, no studies addressing the pharmacological properties of *H.* spp. with experimental designs in in vitro and in vivo models have been published to date on continents other than the Americas and Asia, as shown in Figure 5.

**Figure 6:**
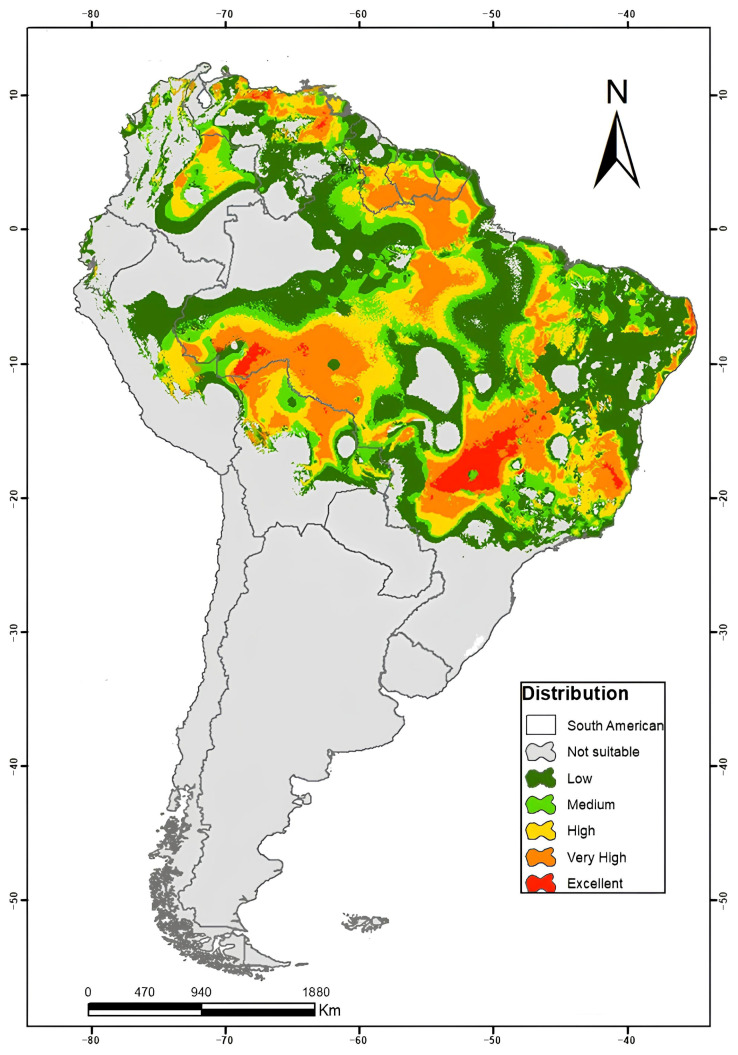
Distribution model of the occurrence map of *H.* spp. in South America, generated using DIVA-GIS software, version 7.5, applying the Bioclim model and the UTM Coordinate System—Horizontal Datum: SIRGAS 2000. Source: The authors.

Occurrence data for *H.* spp. in South America were obtained from multiple biodiversity databases, including the Global Biodiversity Information Facility (GBIF, https://doi.org/10.15468/dl.3u2cz8, accessed on 18 June 2025), SpeciesLink (https://specieslink.net/, accessed on 18 June 2025), and the Brazilian Biodiversity Information System (SiBBr, https://stip.oecd.org/stip/interactive-dashboards/policy-initiatives/2025%2Fdata%2FpolicyInitiatives%2F200002468/, accessed on 18 June 2025), and are presented in Figure 6. A total of 307 occurrence points were used, undergoing a careful curation process to remove geographic inconsistencies such as coordinates located at country centroids or institutional addresses. Distribution modeling of *H.* spp. was performed using DIVA-GIS software, version 7.5, applying the Bioclim model based on current climate prediction data. The Bioclim algorithm, widely used in ecological niche modeling, estimates the potential distribution of species based on bioclimatic envelope limits as described by [59]. The environmental variables used in the modeling were obtained from the WorldClim database, with a spatial resolution of 5 arc-min, which provides high-resolution global climate data.

**Figure 7:**
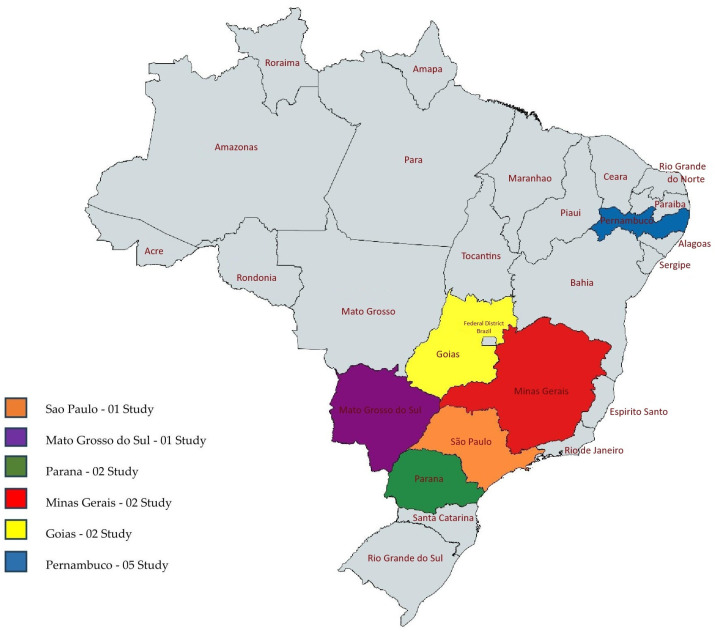
Geographical and quantitative distribution of studies included in this review conducted in Brazil.

**Figure 8:**
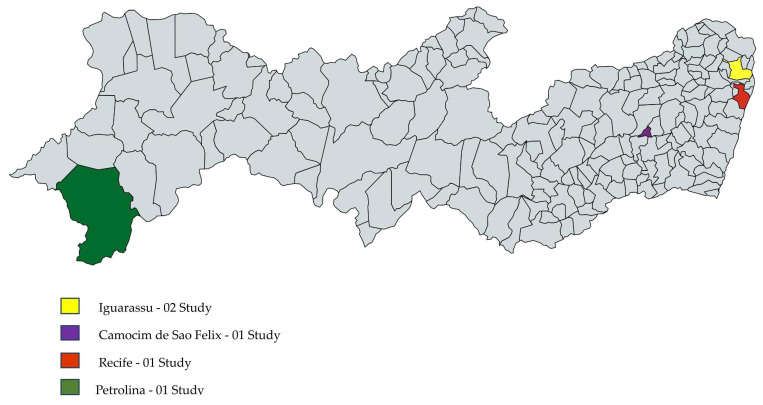
Geographical and quantitative distribution of studies included in this review conducted in the state of Pernambuco, Brazil.

The geographic and quantitative distribution of studies conducted in Brazil of *H.* spp. is presented in Figure 7, while Figure 8 details this distribution for municipalities in the state of Pernambuco, which accounted for the most significant number of studies.

### 2.3. Study Characteristics

The articles included in this systematic review provide evidence of the pharmacological properties of *H.* spp., predominantly through in vitro experimental studies. The extracted data detail the type of study, pharmacological activity, identified compounds, and prominent authors. In total, 12 articles were incorporated into the data synthesis, as presented in Table 2.

Among the studies included in this review, 5 specifically investigated the anti-inflammatory and immunomodulatory effects of *H.* spp. (Table 3). Three investigations demonstrated, through chemical analysis by UPLC-HRMS/MS, the use of *H. courbaril* as an antioxidant in antimicrobial therapy with antibiofilm effect [13]. The essential oil of *H. rubriflora* showed antibacterial and antifungal activities through the constituents E-caryophyllene (36.72 ± 1.05%), Germacrene D (16.13 ± 0.31%), a-humulene (6.06 ± 0.16%), b-elemene (5.61 ± 0.14%), and d-cadinene (3.76 ± 0.07%). The mechanism of antimicrobial action may be related to cell permeabilization and disruption of membrane integrity [12]. Xyloglucans from *H. courbaril* induce the secretion of IL-1, which can be considered an enhancer of tumor elimination [11]. Furthermore, seed-derived polysaccharides were shown to modulate macrophage phagocytic activity and increase NO synthesis [13], indicating immunomodulatory action. Although most evidence derives from acute inflammation models, no study to date has investigated chronic inflammatory pathways or conducted comprehensive in vivo cytokine profiling, and collectively, these results corroborate the traditional use of *Hymenaea* in inflammatory disorders while underscoring its potential for further pharmacological development targeting inflammation.

### 2.4. Chemical Structure

The studies included in this review report diverse pharmacological activities, including anti-inflammatory, antimicrobial, antiproliferative, immunomodulatory, antioxidant, analgesic, antifungal, antibacterial, antiplasmodial, antigenotoxic, antimutagenic, and antirecombinogenic effects, among others. In each study, the identified compounds were described, with their 2D and 3D molecular structures organized alongside their respective SMILES notations. The molecular structures were drawn using the MolView tool, which accesses the PubChem database. Full details are presented in Table 4.

The phenolic compounds and flavonoids listed, including astilbin, rutin, eucryphin, taxifolin, and the procyanidin B2 dimer, exert their anti-inflammatory activities primarily through the mechanism of scavenging reactive oxygen species (ROS) and modulating key signaling pathways, such as the nuclear factor kappa B (NF-κB) pathway. The presence of multiple phenolic hydroxyl groups in their structures confers a potent antioxidant effect, allowing them to neutralize free radicals and, consequently, attenuate oxidative stress—a primary trigger of the inflammatory response. By inhibiting the activation of NF-κB, these compounds prevent the subsequent expression of pro-inflammatory genes, leading to a reduction in the synthesis of mediators such as nitric oxide (NO), prostaglandins, and cytokines (e.g., TNF-α and IL-6). This dual action is particularly evident in studies with astilbin and rutin, which have demonstrated significant suppression of NO production in models of stimulated macrophages [64].

Complementarily, the identified terpenoids, such as caryophyllene oxide [65], lupeol [66], and (E)-caryophyllene [65], exhibit a distinct pharmacological profile by acting as modulators of other molecular targets involved in inflammation. Studies suggest that caryophyllene oxide may mediate its effects through interaction with cannabinoid receptors or by inhibiting cyclooxygenase-2 (COX-2), a central enzyme in prostaglandin production. Lupeol, a pentacyclic triterpene, has been shown to suppress phospholipase A2 activity and histamine release, acting on early stages of the inflammatory cascade. Meanwhile, methylated flavonoids, such as genkwanin, exhibit potentially superior bioavailability, which may amplify their in vivo effects [67]. The synergy between these different classes of compounds—flavonoids, proanthocyanidins, and terpenes—present in *H.* spp. extracts suggests a multifaceted and cooperative anti-inflammatory mechanism, justifying the traditional use of these plants in managing inflammatory conditions.

#### 2.4.1. Immunomodulatory and Anti-Inflammatory Response

Polysaccharide extracts obtained from the seeds of *H. courbaril* demonstrated significant immunomodulatory activity, characterized by the activation of murine peritoneal macrophages and the induction of mediators of the innate immune response [13,68,69]. In in vivo models, a marked increase in peritoneal macrophage counts was observed, exceeding +500% within 24 h after oral administration of the extracts, accompanied by an increase in nitric oxide production in cell cultures, indicating functional activation of these cells [68]. The polysaccharides also induced the dose-dependent synthesis of pro-inflammatory cytokines—IL-1β, IL-6, and TNF-α—suggesting that the bioactive compounds of *H. courbaril* stimulate pattern recognition receptors, possibly TLR2 and TLR4, thereby triggering NF-κB pathway activation and the transcription of genes associated with innate immune responses [13,69].

In parallel, the studies demonstrated that the immunomodulatory activity of *H. courbaril* also involves a regulatory anti-inflammatory effect, characterized by a controlled reduction in the pro-inflammatory response and the modulation of oxidative mediators [13,69]. Enzymatic degalactosylation of the polysaccharides altered, but did not suppress, the production of NO and cytokines, suggesting that minor structural modifications in plant polymers influence the intensity and balance of the immune response [69]. This functional plasticity indicates that the constituents of *H. courbaril* act as fine modulators of inflammation, capable of stimulating defense mechanisms without triggering excessive inflammatory reactions.

#### 2.4.2. Gastroprotection and Antioxidant Activities

The extracts obtained from *H*. spp. exhibited pronounced gastroprotective effects, closely associated with their anti-inflammatory and antioxidant activities. In experimental models, the methanolic extract of *H. stigonocarpa* and the ethanolic extract of *H. martiana* significantly reduced mucosal damage and leukocyte infiltration, with results comparable to those achieved by standard antiulcer drugs such as sulfasalazine and indomethacin [22,70,71]. Administration of *H. martiana* extracts at 200–400 mg/kg decreased peritoneal leukocyte migration up to 90%, indicating a potent inhibition of inflammatory mediators associated with gastric injury, including prostaglandin E_2_, TNF-α, and IL-1β. Similarly, *H. stigonocarpa* extracts restored glutathione levels, reduced malondialdehyde formation, and inhibited myeloperoxidase activity, confirming the attenuation of oxidative stress and lipid peroxidation in gastrointestinal tissues [72].

Enhanced production of phenolic compounds obtained through optimized extraction methods, such as hydroethanolic and ultrasound-assisted extraction, resulted in vigorous radical-scavenging activity, with IC_50_ = 0.71–15.4 µg/mL [73,74]. These findings demonstrate that *H.* spp. protect the gastric mucosa through dual mechanisms: (i) suppression of inflammatory cascades by Cyclooxygenase/Lipoxigenase inhibition, and (ii) enhancement of the endogenous redox defense system, supporting their ethnopharmacological use in the prevention of ulcerative and oxidative gastrointestinal disorders.

#### 2.4.3. Bronchoprotective Effects

Extracts of *H. courbaril* have demonstrated remarkable bronchoprotective and respiratory anti-inflammatory properties in both in vitro and in vivo models. The ethyl acetate fraction of the ethanolic stem bark extract significantly reduced airway hyperresponsiveness, eosinophilia, and neutrophilia in ovalbumin-challenged rats, indicating attenuation of allergic airway inflammation [75]. The same fraction extracted from the stem bark promoted relaxation of tracheal smooth muscle against contractions induced by carbachol and potassium chloride with 95–100% inhibition, an effect attributed to the blockade of voltage-dependent calcium channels and the modulation of intracellular calcium homeostasis. In parallel, the crude extract displayed vigorous antioxidant activity, with IC_50_ = 3.07–5.12 µg/mL, by DPPH assay, suggesting that the reduction in oxidative stress also contributes to the bronchodilatory response [75]. Together, these findings provide the first experimental evidence of *H. courbaril*’s dual role as a respiratory anti-inflammatory and smooth muscle relaxant, supporting its ethnopharmacological use in treating asthma, bronchitis, and allergic airway disorders.

Complementary studies have revealed that the resin of *H. courbaril* acts as a potent 5-lipoxygenase inhibitor, achieving 100% enzyme inhibition at 19 µg/mL, thereby suppressing the synthesis of leukotrienes B_4_, C_4_, and D_4_, which are key mediators of bronchoconstriction and leukocyte recruitment [76]. These molecular and physiological data indicate that *H.* spp. exert bronchodilatory and bronchoprotective effects through integrated mechanisms: (i) inhibition of the 5-lipoxigenase/leukotriene pathway, (ii) modulation of calcium-dependent muscle contraction, and (iii) antioxidant suppression of oxidative and inflammatory stress. Collectively, these actions validate *H. courbaril* as a promising source of natural compounds for respiratory inflammation control and airway protection.

### 2.5. Extracting the Results and Bias Analysis

**Figure 9:**
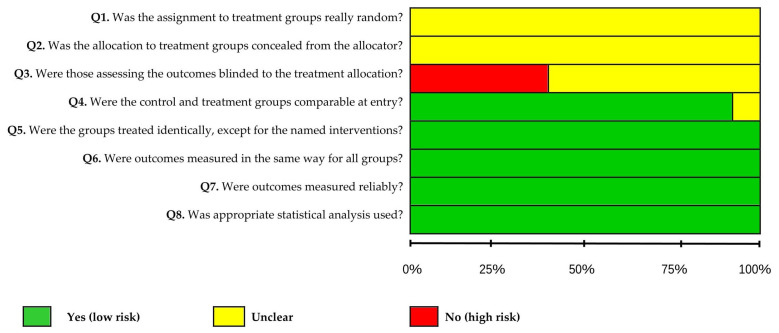
Risk of bias graph: review authors’ judgments on each bias item, expressed as percentages across all included studies.

**Figure 10:**
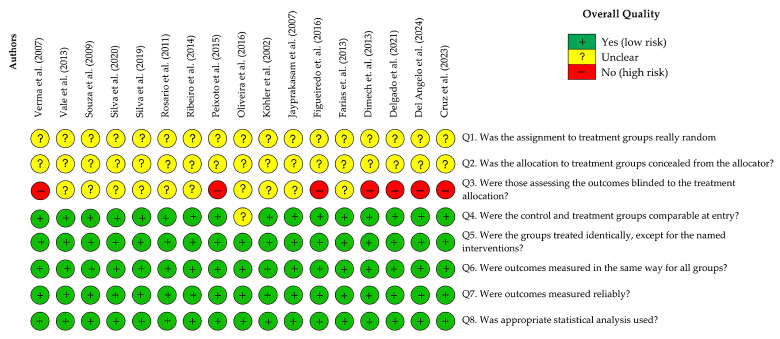
Risk of bias graph and overall quality: authors’ judgments on each bias item for each included study. The corresponding references follow from right to left [4,5,6,7,10,11,12,13,14,20,21,23,24,60,61,62,63].

The results were extracted by screening the titles and abstracts via Mendeley Desktop software (v1.19.8). The full texts of the selected studies were constructed on the basis of the established inclusion and exclusion criteria. Data extraction was carried out according to the analysis of scientific evidence on the pharmacological properties and therapeutic uses of *H.* spp. The bias analysis of the 17 articles included in this review revealed that 94.1% presented a low risk of bias for the questions that addressed topics such as group and treatment. The questions that evaluated treatment groups, measured and reliable results, and appropriate statistical tests indicated a low risk of bias in 100% of the articles. An unclear risk of bias for the questions that addressed random assignment to treatment and treatment concealment of the allocator was identified in 100% of the articles included in this review. This is justified, since 88.2% of the studies are in vitro, and the two in vivo studies included in this review also do not clarify these issues. The item that asks whether those who evaluated the results were blinded to the treatment allocation showed a high risk of bias in 41.2% of the articles. For more information, see Table 5 and Figure 9 and Figure 10.

## 3. Discussion

The deep ecological roots and the long-standing relationship between humans and *H.* spp. are evident in the convergence of traditional knowledge and contemporary pharmacological research. For generations, indigenous and rural communities have employed the bark and sap of these species to treat respiratory ailments, inflammation, and skin wounds [15,62]. Modern scientific investigations increasingly validate these uses, confirming the species’ notable anti-inflammatory, antioxidant, and wound-healing properties. In this context, a systematic analysis of the available pharmacological evidence reveals a promising, albeit still preliminary, scenario regarding the scientific substantiation of its therapeutic applications. Most studies included in this review were in vitro and primarily investigated anti-inflammatory, antimicrobial, antiproliferative, immunomodulatory, and antioxidant activities [6,7,11]. These findings, therefore, partially corroborate the traditional use of *H.* spp. leaves, bark, seeds, and resin, while also demonstrating that the phytochemical composition of its extracts varies according to extraction method, collection region, plant part used, and mode of preparation. For instance, taxifolin derivatives, condensed tannins, and catechins predominate in bark and leaf extracts. In contrast, β-elemene and volatile sesquiterpenes, such as germacrene D, are more frequently found in essential oils [12]. Among the most recurrent bioactive compounds identified are flavonoids, such as quercetin, rutin, and astilbin, along with their derivatives, as well as proanthocyanidins, diterpenes, triterpenes, sterols, and diverse phenolic compounds [5,10,63].

Taken together, these data illustrate that the chemical richness of *H.* spp. constitutes a solid biochemical basis for the species’ multiple biological activities. However, the excellent chemical diversity presented in these studies extends the pharmacological potential of *H.* spp., but highlights a critical gap to be addressed, namely the absence of clear correlations between the metabolites identified and their mechanisms of action in more complex biological models. This chemical versatility directly influences the observed bioactivities, reinforcing the need for reproducibility and standardization in preclinical study models. Beyond these aspects, some studies have demonstrated the antimutagenic and antigenotoxic properties of *H.* spp. extracts, suggesting genomic-level protection and offering prospects for neuroprotective and chemopreventive applications in cancer treatment, although several aspects remain unclear [63]. Moreover, a subset of in vivo studies has reported antiparasitic and analgesic effects [24,62], with antiparasitic potential supported by activity against *Plasmodium falciparum* and *Leishmania amazonensis* [4,60]. Notably, essential oils from *H.* spp. have shown antibiotic potential against resistant bacterial strains, a distinctive result that opens avenues for therapeutic approaches in antimicrobial resistance scenarios [12]. In addition, antimicrobial activity, particularly against *Staphylococcus aureus*, has been consistently observed. However, evidence remains limited regarding whether these effects are bactericidal or bacteriostatic, and whether synergism with conventional antibiotics occurs [11,21]. One study investigating such interactions demonstrated the potentiation of antibiotic efficacy, underscoring an underexplored opportunity in the fight against antimicrobial resistance [12].

Building upon these pharmacological trends, some *H.* spp. have demonstrated immunomodulatory effects, notably through increased nitric oxide production and macrophage stimulation; however, these observations are mostly restricted to isolated cell lines, without further exploration of accessory immune pathways or cytokine profiles [13]. In the context of cytotoxic and antiproliferative activities, terpenes and flavonoids are frequently cited as bioactive constituents. Yet, no study has performed transcriptomic analysis or receptor-binding assays to elucidate the specific intracellular targets involved in tumor suppression [10,23]. Polysaccharides extracted from the seeds of *H.* spp. have also been shown to enhance nitric oxide production and phagocytic capacity, reinforcing the species’ immunostimulatory potential [13]. This review further identified caryophyllene oxide as an active compound with antiproliferative effects in prostate cancer cell lines, though the intracellular mechanisms driving apoptosis remain unexplored [10]. Overall, these immunomodulatory and antiproliferative effects may partially explain the traditional use of these plants in managing infections and inflammatory conditions, suggesting relevant pharmacological potential for inflammatory disease management, albeit still at a preliminary stage. The anti-inflammatory, immunomodulatory, and antioxidant properties, which are mainly from in vitro models, indicate possible adjunctive applications in conditions such as arthritis, dermatitis, or mild systemic inflammatory processes [11,12,13]. Therefore, future clinical trials incorporating inflammatory biomarkers, dose–response assessments, and safety evaluations will be essential to validate these therapeutic applications. However, the absence of methodological standardization and the limited investigation of metabolic pathways hinder comparative analyses across studies. Variations in plant parts used, extraction methods, and lack of systematized quantification of bioactive compounds further complicate data integration [14,23]. Strengthening the interconnection between pharmacological and phytochemical approaches will thus be key to translating traditional knowledge into contemporary therapeutic strategies.

Within this broader pharmacological context, the evidence obtained from immunobiological assays confirms and expands the understanding that the metabolites of *H.* spp. function as fine regulators of immune signaling, integrating both innate and adaptive mechanisms in a coordinated manner [13,68,69]. Rather than merely inducing macrophage activation, the species appears to balance pro- and anti-inflammatory cascades, suggesting a homeostatic regulatory effect in which polysaccharides and secondary metabolites fine-tune early inflammatory events while preventing immune overactivation [68,69]. This dual and self-limiting modulation reflects the behavior of complex phytochemical matrices capable of interacting synergistically with pattern-recognition receptors (TLRs) and intracellular mediators such as NF-κB, thereby harmonizing the production of key immune messengers, including NO, IL-1β, and TNF-α [13,68,70]. Consequently, this coordination among cellular and molecular pathways reinforces the notion of immune homeostasis, in which *Hymenaea* metabolites not only modulate inflammation but also support tissue repair and strengthen endogenous antioxidant defenses [13,68,70].

Extending this concept of integrated regulation, the experimental evidence consolidates the view that *H.* spp. metabolites promote gastric protection through a dynamic interplay of anti-inflammatory and antioxidant pathways, rather than a single pharmacological mechanism. The simultaneous inhibition of COX and LOX activities and the modulation of the NF-κB axis appear to converge toward limiting oxidative and inflammatory damage, thus preserving mucosal integrity [70,71,72]. Such multi-target regulation reflects the capacity of plant-derived compounds to modulate interconnected molecular networks—including eicosanoid biosynthesis, cytokine signaling, and redox balance—rather than acting through isolated targets [70,73]. The resulting reinforcement of endogenous antioxidant defenses, notably through increased GSH availability and reduced lipid peroxidation (MDA, MPO), supports a systemic rebalancing of oxidative homeostasis, which may explain the observed mucosal recovery [74]. Altogether, these interactions suggest that *Hymenaea* metabolites orchestrate complementary enzymatic and redox mechanisms that underlie their traditional antiulcer and gastroprotective reputation, bridging ethnopharmacological use with molecular evidence.

Similarly, the experimental data collectively corroborate the hypothesis that *H.* spp. exert bronchoprotection through the coordination of myorelaxant, anti-inflammatory, and antioxidant mechanisms, reflecting a multi-target pharmacological profile. Rather than acting via a single pathway, the extracts appear to integrate the inhibition of 5-lipoxygenase activity, attenuation of calcium-dependent smooth muscle contraction, and mitigation of reactive oxygen species, all of which converge to alleviate airway hyperresponsiveness [75,76]. This integrative regulation suggests that *H. courbaril* metabolites simultaneously interfere with eicosanoid biosynthesis and intracellular calcium flux, thereby restoring bronchial tone and reducing inflammatory cell recruitment [75,76]. In addition, the potent antioxidant capacity observed in vitro may further enhance these effects by limiting oxidative cascades that perpetuate airway inflammation [75]. Together, these mechanisms outline a coherent model in which *Hymenaea* metabolites confer bronchodilatory and anti-inflammatory protection, providing a biochemical rationale that supports their traditional use in the management of asthma, bronchitis, and related respiratory conditions.

Beyond these pharmacodynamic aspects, metabolomics has emerged as a powerful tool in medicinal plant research, enabling comprehensive characterization of metabolic profiles and identification of biomarkers linked to therapeutic activity. Although still incipient for *H.* spp., promising advances, especially in *H. courbaril*, highlight its value for quality control, mechanism elucidation, and discovery of therapeutic markers [60,61,76]. For species with high chemical variability, this type of approach proves essential to ensuring reproducibility, efficacy, and safety in the development of products based on natural extracts. A study using UPLC-ESI-QTOF-MS/MS characterized phenolic compounds in *H. courbaril* pod residue, revealing proanthocyanidins “dimeric to tetrameric’, taxifolin, and quercetin derivatives, underscoring the pharmacological and biotechnological potential of underused plant byproducts [7]. Comparative analyses of leaves, bark, and seeds identified rutin, luteolin, isorhamnetin, and epigallocatechin with antioxidant and antimicrobial potential [11]. Moreover, metabolomics also reveals the influence of ecological and geographic factors on secondary metabolism, as seen in distinct Cerrado and Amazonian chemotypes of *H. courbaril*, which differ in flavonoid, tannin, and terpene profiles [77,78]. Standardized collection and extraction are essential in ensuring reproducibility and efficacy. Integration of metabolomics with pharmacological bioassays and chemometric tools, PLS-DA, and OPLS allows correlation of specific compounds with biological activities, aiding the identification of quality markers. Additionally, exploring bioactive compounds in pods, bark, and resins supports the circular economy by adding value to agro-industrial waste and promoting sustainable natural product development [61,77].

Finally, despite the advances summarized above, the pharmacological findings in this review, although promising, reveal significant gaps in scientific knowledge regarding *H.* spp. Most available data is derived from in vitro investigations, which, although relevant, do not provide sufficient evidence to support therapeutic applications. Few studies have progressed in animal models, and even fewer have addressed toxicological safety or the pharmacokinetics of *H.* spp. bioactive compounds. A significant limitation is the lack of research elucidating the molecular mechanisms of the action of these compounds; despite frequent reports of tannins, triterpenes, and flavonoids, the metabolic pathways and specific cellular targets remain unclear, hindering therapeutic development. Another critical gap is the absence of clinical evidence, given that no clinical trials or observational studies in humans have been conducted, which makes it challenging to incorporate *H.* spp. into evidence-based practice, despite its broad traditional use. Additionally, methodological heterogeneity, including differences in plant parts studied (bark, seeds, leaves, resins), extraction types (ethanolic, aqueous, essential oils), and chemotypic variation between geographic regions, results in inconsistencies in pharmacological effects and chemical profiles. Ensuring reliability and reproducibility requires standardized extraction methods and comparative analyses. Therefore, future research should prioritize robust animal models, standardized in vivo trials, toxicological and pharmacokinetic evaluation of major bioactive compounds, molecular studies to identify signaling pathways and cellular targets, and human clinical trials assessing efficacy and safety. Ultimately, although unique characterization and extraction protocols for *H.* spp. have been developed, addressing these gaps is essential in translating its therapeutic potential, long recognized in traditional culture, into scientifically validated applications.

## 4. Materials and Methods

**Figure 11:**
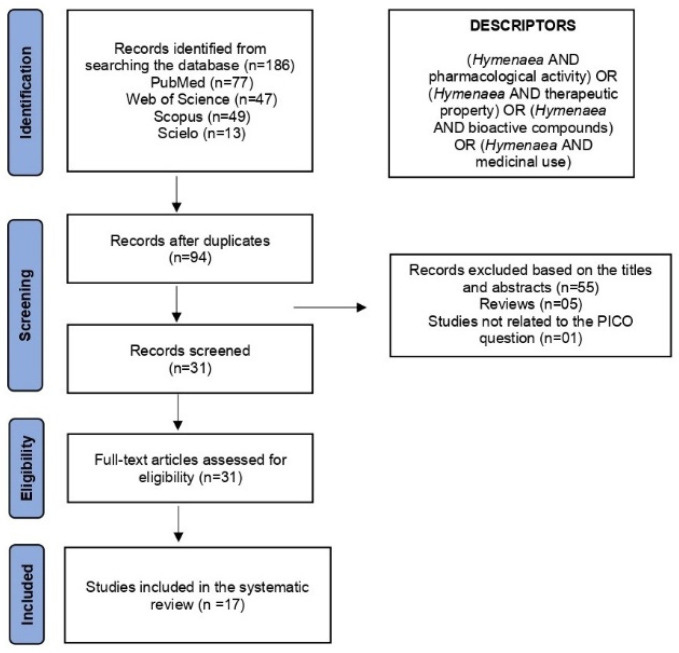
PRISMA flowchart of the selection process for studies on *H.* spp. retrieved from electronic databases. Source: Ref. [79].

This systematic review followed PRISMA recommendations and was not prospectively registered. During the search of the electronic databases, 186 articles were identified: 77 from Medline/PubMed, 47 from Web of Science, 49 from Scopus, and 13 from SciELO. A flowchart of the selection process is shown in Figure 11. Of these, 94 were duplicates. From the remaining 92 records, 55 were excluded based on titles and abstracts that did not meet the inclusion criteria, 5 were literature reviews, and 1 was unrelated to the PICO question of this systematic review. After full-text assessment, 31 articles met the eligibility criteria by addressing, at least partially, the guiding question: “What are the proven pharmacological properties of *H.* spp.?” Ultimately, 17 studies were included in this systematic review.

The review based on the PICO framework was: P (Population): *H.* spp.; I (Intervention): pharmacological use and/or bioactive compounds; C (Comparison): not applicable; O (Outcome): scientifically demonstrated pharmacological properties. Literature searches were conducted up to June 2025, in PubMed, Scopus, Web of Science, and SciELO, selected for their proven high performance in retrieving evidence for systematic reviews [78], with no language or date restrictions; digital translation tools were used when necessary. The core search terms were combined in four groups, namely “*Hymenaea* AND pharmacological activity,” “*Hymenaea* AND therapeutic property,” “*Hymenaea* AND bioactive compounds,” and “*Hymenaea* AND medicinal use”, and explored in multiple permutations. However, the initial strategy did not restrict outcomes to inflammation, screening, and extraction; it prioritized studies reporting anti-inflammatory, immunomodulatory, or inflammation-related activities. All records were imported into Mendeley Desktop (v1.19.8) for duplicate removal, followed by title/abstract screening against predefined inclusion criteria, and full-text assessment of potentially eligible studies.

### 4.1. Inclusion/Exclusion Criteria and Data Extraction

The inclusion criteria were: (1) original research articles investigating the pharmacological properties and/or therapeutic uses of any *H.* spp.; (2) publications in any language, with no restriction on publication date; (3) studies employing in vitro, in vivo, or clinical trial designs; and (4) full-text availability. Exclusion criteria comprised: (1) review articles; (2) studies focused solely on taxonomy, ecology, or ethnobotany; (3) conference abstracts, editorials, letters to the editor, or other brief scientific communications; (4) articles whose full text could not be obtained despite repeated attempts; and (5) articles failing to meet the inclusion criteria upon full-text review. Extracted data included bibliographic information, study type, pharmacological activity, identified compounds, and reported clinical or therapeutic applications.

### 4.2. Data Visualization and Risk of Bias

VOSviewer^®^ software (version 1.6.20 for Windows) was used to construct and visualize co-occurrence networks of terms retrieved from the bibliometric search, as well as the primary authors and publication years [80]. The risk of bias for the studies included in this systematic review was independently assessed by two reviewers (JBC and ABS) following the criteria proposed by the Joanna Briggs Institute [81], and in cases of disagreement, a third reviewer (JOF) was consulted. The original tool contains 10 questions, two of which were excluded because they were not applicable to bias assessment in in vitro experimental studies. The following questions were used: (1) Was the assignment to treatment groups truly random? (2) Was the allocation to treatment groups concealed from the allocator? (3) Were outcome assessors blinded to the treatment allocation? (4) Were the control and treatment groups comparable at baseline? (5) Were the groups treated identically, except for the intervention under investigation? (6) Were the outcomes measured in the same way for all groups? (7) Were the outcomes measured reliably? (8) Was an appropriate statistical analysis applied? A “Yes” response, when supported by sufficient information, was classified as indicating low risk of bias, whereas a “No” response or insufficient information was classified as high risk of bias. The “Unclear” category was applied when the available information was inadequate to determine whether the risk of bias was high or low.

## 5. Conclusions

The present study collected and critically analyzed the available literature on the pharmacological properties of *H.* spp., revealing promising therapeutic potential supported by several in vitro and in vivo studies. Anti-inflammatory, antimicrobial, antioxidant, and immunomodulatory activities are frequently reported and associated with bioactive compounds such as flavonoids, proanthocyanidins, triterpenes, and sesquiterpenes, which partially corroborate their traditional applications. However, the current evidence lacks methodological robustness, progression to standardized in vivo models, and especially clinical validation. Gaps such as the absence of clinical trials, variability in extraction methods, plant parts used, and the scarcity of investigations into molecular mechanisms limit the translation of these findings into evidence-based therapeutic practice. In this scenario, expanding interdisciplinary efforts that integrate clinical, phytochemical, and pharmacological data through standardized research protocols is urgent. Metabolomic approaches emerge as a strategic tool by enabling comprehensive compound profiling and correlation with pharmacological responses. The integration of metabolomics with conventional pharmacological models may foster standardization, reproducibility, and scientific validation, leading toward safe and effective therapeutic applications.

Furthermore, the anti-inflammatory and immunomodulatory activities repeatedly observed, mainly in vitro, highlight *H.* spp. as promising candidates for adjunctive strategies in managing inflammatory conditions. Although translational studies remain scarce, current evidence justifies further investigation into oxidative stress and cytokine modulation. Ultimately, *H.* spp. represents a bridge between environmental history, cultural heritage, and pharmacological promise. The ongoing validation of these ancestral uses by modern pharmacology paves the way for innovative therapies and contemporary validation of ancient knowledge.

## Figures and Tables

**Table 1:** Ethnobotanical, pharmacological, and experimental details of *H.* spp. identified in a systematic literature search.

Name of the Species	Traditional Use	Traditional Preparation for Consumption	Dosage of Consumption	Type of Study	Experimental Details	Authors
*H. cangaceira*	Infections, inflammation, and pain	Not reported	Safe up to 5000 mg/kg (oral, mice)	Experimental—in vitro	Demonstrated antioxidant activity and biofilm inhibitory activity against *Staphylococcus aureus*	[47]
*H. courbaril*	Antioxidant, energetic, and anti-inflammatory	Licking, roasting, grinding, and decoction	Not reported	Experimental—in vitro	Demonstrated antitumor activity in PC-3 human prostate cancer cells and antimicrobial activity against bacteria and fungi	[48,49]
*H. eriogyne*	Infections; antioxidant	Not reported	Not reported	Experimental—in vitro	Demonstrated antioxidant activity (DPPH, ABTS assays) and immunological activity in murine peritoneal macrophages	[50,51]
*H. martiana*	inflammation, pain, infections; popular use as tea or ethanol extracts	Macerated leaves, fruits, and seeds in 95% ethanol	Not reported	Experimental—in vivo	Demonstrated ecophysiological responses in young plants	[6]
*H. oblongifolia*	Not reported	Not reported	Not reported	Observational field study and experimental—in vitro	Demonstrated reproductive traits, phytochemical composition, and antioxidant activity	[52,53]
*H. parvifolia*	Astringent; analgesic	Ethanolic extract of the bark	Not reported	Experimental—in vitro	Demonstrated antimicrobial activity against clinical bacteria	[54]
*H. rubriflora*	Respiratory, intestinal, viral, and fungal infections	Infusions and mixtures with leaves, roots, and stems	Not reported	Experimental—in vitro	Demonstrated toxicity and antioxidant activity in B16F10 melanoma cells	[12]
*H. stigoncarpa*	Gastric pain, ulcers, diarrhea, inflammation, and environmental adsorbent	Decoction of the stem bark, use of the fruit pulp, ground fruit peel, natural or treated with HNO3 and NaOH	Not reported	Experimental—in vitro Experimental—in vivo	Demonstrated phytochemical profile and antioxidant activity using DPPH free radical assay, immunological activity using murine peritoneal macrophages, antimicrobial activity against *Staphylococcus aureus* and other pathogens, and ecophysiological responses in seedlings	[5,55,56,57]
*H. velutina*	Not reported	Not reported	Not reported	Experimental—in vivo	Demonstrated pharmacological and veterinary effects in goats	[15]
*H. verrucosa*	Not reported	Not reported	Not reported	Experimental—in vitro	Demonstrated antifungal activity against *Cryptococcus* dermatophytes	[58]

**Table 2:** Characteristics of in vitro and in vivo studies on the pharmacological activities and bioactive compounds of *H.* spp.

Study Type	Pharmacological Activity	Identified Compounds	Authors
In vitro	Antioxidant, antimicrobial and anti-biofilm against *Staphylococcus aureus*	Chrysoeriol-7-O-neohesperidoside, isorhamnetin-3-O-glucoside, 3,7-di-O-methylquercetin, rutin, myricetin, luteolin, epigallocatechin-3-O-gallate-3′-O-glucuronide, quercetin-3-O-glucoside, isorhamnetin, kaempferol-3-O-rutinoside, sucrose	[11]
In vitro	Antiproliferative; apoptotic	Caryophyllene oxide, (1R,7S,E)-7-isopropyl-4,10-dimethylcyclodec-5-enol, γ-sitosterol	[10]
In vitro	Antimicrobial against Gram-positive bacteria, especially *Staphylococcus aureus*	Terpenes, coumarins, flavonoids, condensed tannins, astilbin	[21]
In vitro	Antioxidant; photoprotective	Flavonoids	[5]
In vitro	Antiplasmodial activity against *Plasmodium falciparum*	Flavones derived from apigenin, linoleic acid, and α-linolenic acid	[60]
In vivo	Antibacterial activity against *Staphylococcus aureus*, reduction in somatic cell count, and colony-forming units in mastitis cases	Flavonoids, steroids, terpenoids	[61]
In vitro	Antileishmanial activity against *Leishmania amazonensis*	Tannins, flavonoids, triterpenes, steroids, lupcol, betulinic acid, verbascoside	[4]
In vitro	Antimicrobial; antioxidant	Astilbin, taxifolin derivatives, rutin, catechin derivatives, condensed tannins, terpenes/steroids, saponins, cinnamic derivatives, ellagic acid	[20]
In vitro	Antimicrobial, antibacterial, antifungal, and synergistic effects with antibiotics	E-caryophyllene, germacrene D, α-humulene, β-elemene, δ-cadinene	[12]
In vitro	Antifungal activity against dermatophytes *Trichophyton rubrum, T. mentagrophytes, Microsporum canis* and *Cryptococcus neoformans*	Triterpenoids, glycosides	[62]
In vivo	Antinociceptive, central and peripheral mechanisms with potential opioid and adenosine receptor involvement	Alkaloids, flavonoids, glycosides, tannins, saponins, lapachone	[24]
In vitro	Antigenotoxic, antimutagenic, antirecombinogenic, anticlastogenic, antianeugenic and antioxidant	Tannins, flavonoids, procyanidins, essential oils, terpenes	[63]

**Table 3:** Studies on anti-inflammatory and immunomodulatory activity of *H.* spp.

Study Type	Pharmacological Activity	Identified Compounds	Authors
In vitro	Antioxidant, antiproliferative, anti-inflammatory, antimicrobial, anti-elastase and anti-collagenase	Procyanidin dimers, trimers, and tetramers derived from quercetin and taxifolin	[7]
In vitro	Antioxidant, anti-inflammatory, and wound-healing	Tannins, flavonoids, sesquiterpene lactones	[14]
In vitro	Antimicrobial, antinociceptive, analgesic, anti-inflammatory, antioxidant	Astilbin, eucrifin, engelitin, taxifolin, triterpenes, steroids, anthracene derivatives, monoterpenes, diterpenes, naphthoquinones, saponins	[6]
In vitro	Inhibition of COX-1 and COX-2 and inhibition of lipid peroxidation	Crotomachlin, labd-13E-en-8-ol-15-oic acid, labd-13E-en-8-ol-15-oic acid methyl ester, labdanolic acid, (13E)-labda-7,13-dien-15-oic acid, labd-8(17),13E-dien-15-oic acid, spathulenol	[23]
In vitro	Immunomodulatory activity via macrophage stimulation	Xyloglucans from seeds of *Copaifera langsdorffii*, *H. courbaril*, *Tamarindus indica*	[13]

**Table 4:** SMILES codes, 2D and 3D chemical structures of compounds, and authors.

SMILES	2D Chemical Structure	3D Chemical Structure	Author
COc1 COc1cc(O)c2c(cc(=O)oc2c1O[C@H]3OC(C(O[C@H]4O[C@@H](C)[C@@H](O)[C@H](O)[C@H]4O)C(O)C(O)C3O)CO)O Chrysoeriol-7-O-neohesperidoside	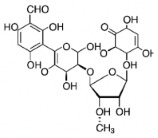	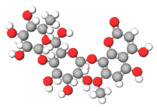	[11]
C1C(C(OC1°C2C(C(C(C(O2)CO)O)O)O)O)OC3C(C(C(C(O3)CO)O)O)O Procyanidin B2 dimer	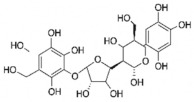	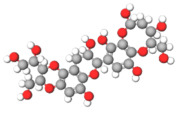	[7,63]
CC1=CCC2C(C1CCC2(C)C)C3CO3 Caryophyllene oxide	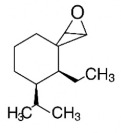	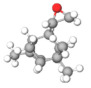	[10]
C[C@H]1[C@@H]([C@H]([C@H]([C@@H](O1)O[C@@H]2[C@H](OC3=CC(=CC(=C3C2=O)O)O)C4=CC(=C(C=C4)O)O)O)O)O Astilbin	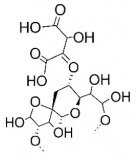	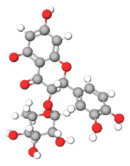	[21,61]
C1=CC=C(C=C1)C2=CC(=O)C3=CC=CC=C3O2 Flavonoid basic skeleton	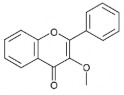	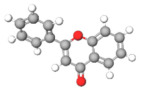	[5,14]
CC1CCC2C(C1)CCC3C2CCC4(C3CCC4=O)O Crotomachlin	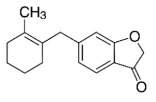	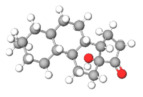	[23]
COC1=CC=C(C=C1)C2=CC(=O)C3=CC=CC=C3O2 Genkwanin	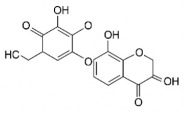	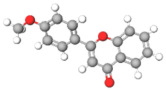	[60]
COC1=CC(=C(C=C1)O)C2=CC(=O)C3=C(O2)C=CC(=C3O)O Engelitin	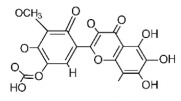	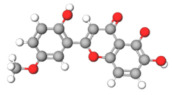	[6]
C1=CC(=C(C=C1C2C(=O)C3=C(C=C(C=C3O2)O)O)O)O Taxifolin	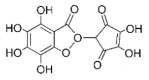	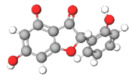	[6]
C=C(C)C1CCC2(C1CCC3C2CCC4C3(CCC(C4)(C)O)C)C Lupeol	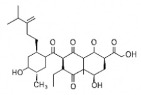	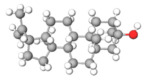	[4]
Polymer β-D-glucose with xylose branches (does not have unique SMILES due to polymer complexity)	Not reported	Not reported	[13]
C1=CC(=C(C=C1C2=CC(=O)C3=C(O2)C=C(C=C3O)O)O)O[C@@H]4[C@H]([C@@H]([C@H](O[C@H]5O[C@@H]([C@H]([C@@H](O5)C)O)CO)O4)O)O Rutin	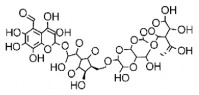	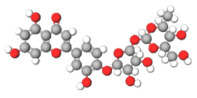	[20]
C=C1CCC2C(C1)CCC3C2CCC3(C)C (E)-Caryophyllene	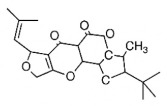	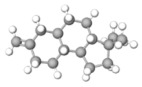	[12]
C1=CC(=C(C=C1C2=CC(=O)C3=C(O2)C=C(C=C3O)O)O)O[C@@H]4O[C@H]([C@@H]([C@H](O4)C)O)O Eucryphin	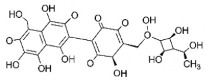	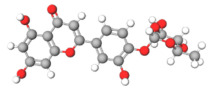	[61]
CC1=CC2=C(C=C1)C(=O)C=CC2=O Lapachone	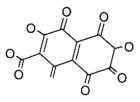	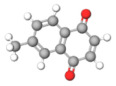	[24]

**Table 5:** Risk of Bias Assessment by Bias Type and Domain in the Included Studies.

	Authors		
	[11]	[7]	[10]
Q1. Was the assignment to treatment groups truly random?	Unclear	Unclear	Unclear
Q2. Was the allocation to treatment groups concealed from the allocator?	Unclear	Unclear	Unclear
Q3. Were those assessing the outcomes blinded to the treatment allocation?	No	No	No
Q4. Were the control and treatment groups comparable at entry?	Yes	Yes	Yes
Q5. Were the groups treated identically, except for the named interventions?	Yes	Yes	Yes
Q6. Were outcomes measured in the same way for all groups?	Yes	Yes	Yes
Q7. Were outcomes measured reliably?	Yes	Yes	Yes
Q8. Was an appropriate statistical analysis used?	Yes	Yes	Yes
	[21]	[14]	[5]
Q1. Was the assignment to treatment groups truly random?	Unclear	Unclear	Unclear
Q2. Was the allocation to treatment groups concealed from the allocator?	Unclear	Unclear	Unclear
Q3. Were those assessing the outcomes blinded to the treatment allocation?	No	Unclear	No
Q4. Were the control and treatment groups comparable at entry?	Yes	Yes	Yes
Q5. Were the groups treated identically, except for the named interventions?	Yes	Yes	Yes
Q6. Were outcomes measured in the same way for all groups?	Yes	Yes	Yes
Q7. Were outcomes measured reliably?	Yes	Yes	Yes
Q8. Was appropriate statistical analysis used?	Yes	Yes	Yes
	[23]	[60]	[6]
Q1. Was the assignment to treatment groups truly random?	Unclear	Unclear	Unclear
Q2. Was the allocation to treatment groups concealed from the allocator?	Unclear	Unclear	Unclear
Q3. Were those assessing the outcomes blinded to the treatment allocation?	Unclear	Unclear	Unclear
Q4. Were the control and treatment groups comparable at entry?	Yes	Yes	Unclear
Q5. Were the groups treated identically, except for the named interventions?	Yes	Yes	Yes
Q6. Were outcomes measured in the same way for all groups?	Yes	Yes	Yes
Q7. Were outcomes measured reliably?	Yes	Yes	Yes
Q8. Was an appropriate statistical analysis used?	Yes	Yes	Yes
	[61]	[4]	[13]
Q1. Was the assignment to treatment groups truly random?	Unclear	Unclear	Unclear
Q2. Was the allocation to treatment groups concealed from the allocator?	Unclear	Unclear	Unclear
Q3. Were those assessing the outcomes blinded to the treatment allocation?	No	Unclear	Unclear
Q4. Were the control and treatment groups comparable at entry?	Yes	Yes	Yes
Q5. Were the groups treated identically, except for the named interventions?	Yes	Yes	Yes
Q6. Were outcomes measured in the same way for all groups?	Yes	Yes	Yes
Q7. Were outcomes measured reliably?	Yes	Yes	Yes
Q8. Was an appropriate statistical analysis used?	Yes	Yes	Yes
	[20]	[12]	[62]
Q1. Was the assignment to treatment groups truly random?	Unclear	Unclear	Unclear
Q2. Was the allocation to treatment groups concealed from the allocator?	Unclear	Unclear	Unclear
Q3. Were those assessing the outcomes blinded to the treatment allocation?	Unclear	Unclear	Unclear
Q4. Were the control and treatment groups comparable at entry?	Yes	Yes	Yes
Q5. Were the groups treated identically, except for the named interventions?	Yes	Yes	Yes
Q6. Were outcomes measured in the same way for all groups?	Yes	Yes	Yes
Q7. Were outcomes measured reliably?	Yes	Yes	Yes
Q8. Was an appropriate statistical analysis used?	Yes	Yes	Yes
	[63]	[24]	
Q1. Was the assignment to treatment groups truly random?	Unclear	Unclear	
Q2. Was the allocation to treatment groups concealed from the allocator?	Unclear	Unclear	
Q3. Were those assessing the outcomes blinded to the treatment allocation?	Unclear	No	
Q4. Were the control and treatment groups comparable at entry?	Yes	Yes	
Q5. Were the groups treated identically, except for the named interventions?	Yes	Yes	
Q6. Were outcomes measured in the same way for all groups?	Yes	Yes	
Q7. Were outcomes measured reliably?	Yes	Yes	
Q8. Was an appropriate statistical analysis used?	Yes	Yes	

## Data Availability

No new data were created or analyzed in this study. Data sharing is not applicable to this article.
